# Bioinspired All-Fibrous Directional Moisture-Wicking Electronic Skins for Biomechanical Energy Harvesting and All-Range Health Sensing

**DOI:** 10.1007/s40820-023-01028-2

**Published:** 2023-03-02

**Authors:** Chuanwei Zhi, Shuo Shi, Shuai Zhang, Yifan Si, Jieqiong Yang, Shuo Meng, Bin Fei, Jinlian Hu

**Affiliations:** 1 Department of Biomedical Engineering, City University of Hong Kong, Hong Kong S.A.R, 999077 China; 2 Institute of Textiles and Clothing, The Hong Kong Polytechnic University, Hong Kong S.A.R, 999077 China; 3 City University of Hong Kong Shenzhen Research Institute, Shenzhen, 518057 People’s Republic of China

**Keywords:** Bioinspired, Electrospinning, Electronic skin, Directional moisture wicking, MXene

## Abstract

**Highlights:**

- Bioinspired directional moisture-wicking electronic skin (DMWES) was successfully realized by surface energy gradient and push–pull effect via the design of distinct hydrophobic-hydrophilic difference.
- The DMWES membrane showed excellent comprehensive pressure sensing performance with high sensitivity and good single-electrode triboelectric nanogenerator performance
- The superior pressure sensing and triboelectric performance enabled the DMWES for all-range healthcare sensing, including accurate pulse monitoring, voice recognition, and gait recognition.

**Abstract:**

Electronic skins can monitor minute physiological signal variations in the human skins and represent the body’s state, showing an emerging trend for alternative medical diagnostics and human–machine interfaces. In this study, we designed a bioinspired directional moisture-wicking electronic skin (DMWES) based on the construction of heterogeneous fibrous membranes and the conductive MXene/CNTs electrospraying layer. Unidirectional moisture transfer was successfully realized by surface energy gradient and push–pull effect via the design of distinct hydrophobic-hydrophilic difference, which can spontaneously absorb sweat from the skin. The DMWES membrane showed excellent comprehensive pressure sensing performance, high sensitivity (maximum sensitivity of 548.09 kPa^−1^), wide linear range, rapid response and recovery time. In addition, the single-electrode triboelectric nanogenerator based on the DMWES can deliver a high areal power density of 21.6 µW m^−2^ and good cycling stability in high pressure energy harvesting. Moreover, the superior pressure sensing and triboelectric performance enabled the DMWES for all-range healthcare sensing, including accurate pulse monitoring, voice recognition, and gait recognition. This work will help to boost the development of the next-generation breathable electronic skins in the applications of AI, human–machine interaction, and soft robots. 
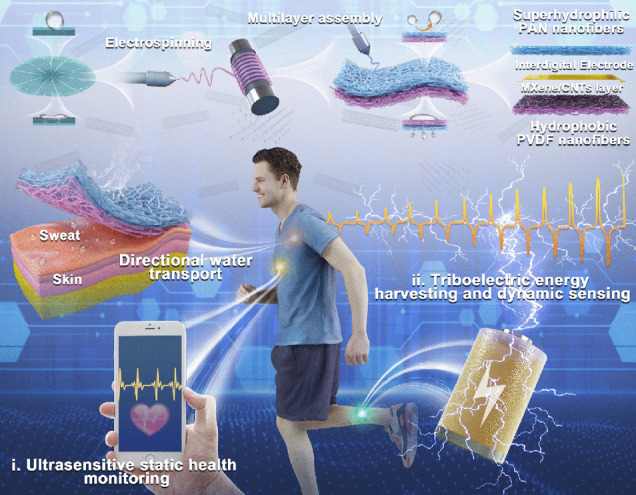

**Supplementary Information:**

The online version contains supplementary material available at 10.1007/s40820-023-01028-2.

## Introduction

Wearable bioelectronics can detect and quantify the physiological data generated by human motions and activities, attracting worldwide research and commercial attention [1–3]. Traditionally designed rigid electrodes are usually attached to the skin or organs through the tapes, clips, or others, resulting in incompatible contact with human body, and distorted or highly noisy physical signals [4]. Herein, recent studies have made great progress in flexible electronic skins with the characteristics of lightweight, ultra-thinness, and comfortability in the applications of personal customized health management, man–machine interface, and artificial intelligence (AI) [5–8]. Nevertheless, many flexible bioelectronics with good acquisition performance were fabricated by impermeable polymer membranes or gels, which hindered the gas or moisture exchange between human skin and ambient environment and resulted in the interference of externally induced thermal or moisture effects [9–11]. Long time contact will also bring about sweat deposition, leading to uncomfortable wearing experience and acquisition signal distortion [12, 13].

Currently, electrospinning technology has developed rapidly in the large-scale manufacturing of fibrous membranes [14, 15]. The nanofiber networks fabricated by electrospinning exhibit high specific surface area, good flexibility and different pore size, rendering it a fascinating candidate for highly sensitive and breathable electronic skins [16–19]. Recent studies have tried to develop high-performance monofunctional pressure sensors or nanogenerators by nanofibrous membrane, in which the nanofibers showed good performance in conductivity or energy harvesting in specific applications [20–23]. In addition, many studies through electrospun fibrous membranes were concentrated on monotonically hydrophilic or hydrophobic architectures, leaving a humid-state skin and limited moisture transfer from the body and electronics, and causing skin discomfort and even inflammation [24–26]. However, with the development of electronic textiles, the large-scale practical application of electronic skins should take its versatility, wearable comfort, and energy-saving performance into account. Therefore, developing moisture-permeable multifunctional electronic skins for physiological monitoring and biomechanical energy harvesting in a single device is of great importance.

MXene is a new type of 2D materials formed by chemically exfoliating the bulk MAX crystalline structure and is normally represented by the formula M_*n*+1_X_*n*_T_X_ (*n* = 1, 2 or 3), where M denotes transition metals (such as Ti, Cr and V), X denotes carbon and/or nitrogen, and T_X_ is surface functional groups (hydroxyl, oxygen, or fluorine terminals). MXene has good metallic conductivity, thermal conductivity, and high surface area [27]. Based on this, several smart electronic skins composed of Ti_3_C_2_T_x_ and their hybrids were designed to realize attractive piezoresistive sensing properties such as low detection limit, high sensitivity, and long stability [28–30]. However, the exceptionally high conductivity and interlayer restacking resulted from hydrogen bonding and van der Waals force give rise to extremely minute changes in the conductive path under tiny pressure and thus, low sensitivity [31]. Due to the low mechanical strength, the long-term deformation and structure integrity cannot be guaranteed under high pressure. In order to achieve the high-performance devices, stable interfaces in different MXene-based hybrid nano-/micro-structures have been constructed in recent years by the design of the heterogeneous interface interactions [32, 33]. Besides, traditional electronic skins and biosensors based on MXenes generally were fabricated by the airproof composites membranes, which may result in uncomfortable wearable experience, microorganism breeding, and skin inflammation [34, 35]. The breathable, stretchable, and thin substrate will improve the perception about micro-deformations of MXene-derived sensing materials.

Nature always offers vast knowledge to humans for the design of materials and devices for controllable wettability [36, 37]. Through the bioinspired design, advanced materials mimicking the functions and nanostructures of living organisms, such as lotus leaf and Namib desert beetle, have been proved to have the capability of directional water transportation by imitating the Janus interface and asymmetric wettability [38–40]. In this regard, we presented the “three birds with one stone” design of bioinspired electronic skin with directional water transfer properties, which was further developed for biomechanical energy harvesting and healthcare sensing. The electronic skin with heterogeneous wettability was comprised of a hydrophobic layer of CNT-modified PVDF nanofibers and a hydrophilic layer of PAN nanofibers sandwiched with electrospraying MXene/CNTs conductive ink. Unidirectional moisture transfer was successfully realized by surface energy gradient and push–pull effect induced by the distinct hydrophobic-hydrophilic difference, which can spontaneously absorb sweat from the skin and ensure bioelectrical signal stability. The directional moisture-wicking electronic skin (DMWES) showed superior static sensing properties, high sensitivity in wide pressure range (maximum sensitivity of 548.09 kPa^−1^), and rapid response/recover time (28.4/39.1 ms). In addition, the single-electrode triboelectric nanogenerator (STENG) based on the DMWES can deliver a high areal power density of 21.6 µW m^−2^ and good cycling stability when applied to high pressure energy harvesting. The superior pressure sensing and triboelectric mechanism enabled the electronic skin for all-range healthcare sensing to monitor pulse accurately, detect gestures and recognize voice visually. This work lays the foundation for the development of the next-generation breathable self-powered electronic skins with high sensitivity, large operating range, and multiple working modes.

## Experimental Section

### Materials

Polyvinylidene fluoride (PVDF), Polyacrylonitrile (PAN), Sodium dodecyl sulfonate (SDS), Lithium chloride (LiCl), Acetone, and Dimethylformamide (DMF) were provided by Shanghai Aladdin Bio-Chem Technology Co., Ltd. All chemicals in experiments were used as received without any treatment. Carboxylated carbon nanotube (CNT) was provided by Nanjing XF Nano Co. Ltd., China (the average diameter is 5–15 nm, and the length is 10–30 μm). Ti_3_AlC_2_ powders were purchased from Jilin 11 Technology Co. Ltd., China.

### Preparation of Colloidal Ti_3_C_2_T_x_ MXene Solution

First of all, 10 mL HCl (9 M) was used to dissolve 1 g LiF at ambient environment. Secondly, Ti_3_AlC_2_ powders (1 g) were gradually added into the above LiF/HCl aqueous solution. Then, the above solution was moved into a Teflon autoclave for 24 h at the temperature of 60 ℃ [41]. After cooling down, the obtained product was subjected to sequential washing of 3 M HCl, then DI water until the pH = 7. Ultimately, the obtained black jelly was ultrasonicated in DI water for 2 h under argon protection and centrifuged at 4,000 rpm. The concentration of the colloidal Ti_3_C_2_T_x_ nanosheets solution was ~ 8 mg mL^−1^. The corresponding TEM image of Ti_3_C_2_T_x_ nanosheets is shown in Fig. S1.

### Preparation of MXene/CNTs Conductive Ink

Carboxylated CNTs (30 mg) was dispersed in 20 mL alcohol/water solution (5/5, v/v). Then, the CNTs solution dispersion was added to the Ti_3_C_2_T_x_ MXene nanosheets colloidal solution (20 mL). To achieve a stable solution ink, the solution was ultrasonicated for 30 min and stirred for 6 h. The corresponding optical image and solution conductivity, TEM image of the MXene/CNTs ink are shown in Figs. S2 and S3, respectively. The solution conductivity of the MXene/CNTs ink was 9.22 μS cm^−1^.

### Preparation of Directional Moisture-Wicking Electronic Skin (DMWES)

Firstly, 3 g PVDF and 0.1 g carboxylic CNTs were dissolved and stirred in 16.9 g DMF/Acetone mixed solution (3/2, wt/wt) with 0.2 wt% LiCl addition. CNT-modified PVDF (C-PVDF) nanofibers membrane as the hydrophobic layer was achieved by electrospinning technique. Secondly, the MXene/CNTs conductive ink was electrosprayed onto the C-PVDF nanofibers. Thirdly, 2 g PAN was dissolved and stirred in 18 g DMF solution with 0.2 wt% SDS addition. Hydrophilic PAN nanofibers membrane as the top layer was achieved by electrospinning onto the electrospraying membrane. During electrospinning and electrospraying processes, the positive voltage and feeding rate were set as 25 kV and 0.05 mm min^−1^, respectively. The tip-to-collector distance was set as 15 cm. The sensing layer was modulated by the electrospraying time of MXene/CNTs conductive ink (electrospraying time for 2, 6, and 10 h, respectively, for DMWES-1, DMWES-2, and DMWES-3).

### Characterization

The chemical structure and groups were characterized by a PerkinElmer FT-IR Spectrometer. X-ray diffraction (XRD) was performed by a D2 Phaser XE-T X-ray diffractometer system, and the scan rate and range of 2θ were set as 5^°^ min^−1^ and 5^°^–70^°^, respectively. X-ray photoelectron spectroscopy (XPS) analysis was achieved by the PHI Model 5802 spectrometer. The surface resistance of the nonwoven membrane was measured by the digital four-probe tester. The morphology was observed by utilizing scanning electron microscope (FEI Quanta 250 e-SEM) and transmission electron microscope (TEM, Philips CM20). For the pressure sensing tests, conductive Cu tapes were adhered onto two electrodes of the interdigital electrode of DMWES, and the DMWES was encapsulated by the medical grade adhesive tape on the top side to ensure to fit skin. The electromechanical property of DMWES was tested using a high-frequency piezoelectric actuator equipped with a digit sourcemeter (Tektronix, 2612B). For the single-electrode TENG tests, the conductive Cu tape was adhered onto the MXene/CNTs layer of DMWES. The output performance of DMWES and force setting was conducted by an electrometer (Keithley 6514) and a linear motor (LinMot E1100), respectively. All the volunteers, two of the authors, participated with informed consent.

## Results and Discussion

### Fabrication and Characterization of DMWES Membrane

**Fig. 1:**
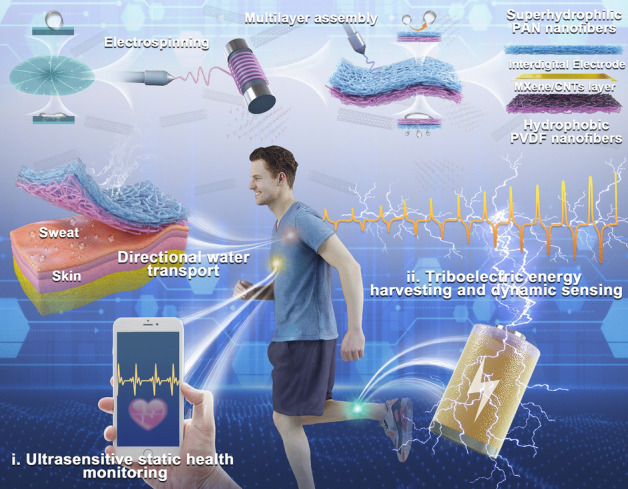
Schematic illustration of the fabrication and application of the DMWES membrane

The animals and plants generally exhibit lots of fascinating functions to adapt to the environment. For example, the Janus wettability of the lotus leaf can keep wet on the hydrophilic side while keep dry on the hydrophobic side. Given this, we developed the multifunctional electronic skin inspired by Janus wettability. The preparation procedure of the directional moisture-wicking electronic skin (DMWES) is demonstrated in Fig. 1. The DMWES was fabricated in three steps. Firstly, a thin and hydrophobic layer of carboxylic CNTs modified PVDF (C-PVDF) nanofibers was electrospun onto the aluminum foil as the bottom layer close to the skin, which can ensure small wetting area and low sweat absorption. Secondly, the MXene/CNTs conductive ink was electrosprayed onto the C-PVDF nanofibers layer. Electrospraying is conducive to control the spraying time and thickness, and therefore, to preserve the porous nature of the fiber substrate with thin spraying layers. The existence of the carboxylic CNTs helps to promote the formation of interaction among C-PVDF, MXenes, and CNTs, avoiding the mutual stacking of MXene lamellae and playing the role of bridging between MXenes. Lastly, a thick and superhydrophilic outer protective layer of PAN nanofibers was constructed by electrospun PAN precursor solution onto the C-PVDF/MXene-CNTs layer. The DMWES effectively pulls the sweat away from the skin and sensing layer, which can evaporate it quickly with wider wetting area and ensure the stable bioelectric signals acquisition. Herein, the constructed DMWES can ensure accurate all-over physiological monitoring and achieve the biomechanical energy harvesting by the single-electrode triboelectric mechanism. When the DMWES functions as the pressure sensor, the MXene/CNTs layer here acts as the conductive sensing layer to perceive the pressure-induced structure change and then, convert into the electrical signal by the interdigital electrode. While the DMWES functions as the STENG, the MXene/CNTs layer performs as the charge acquisition electrode.

**Fig. 2:**
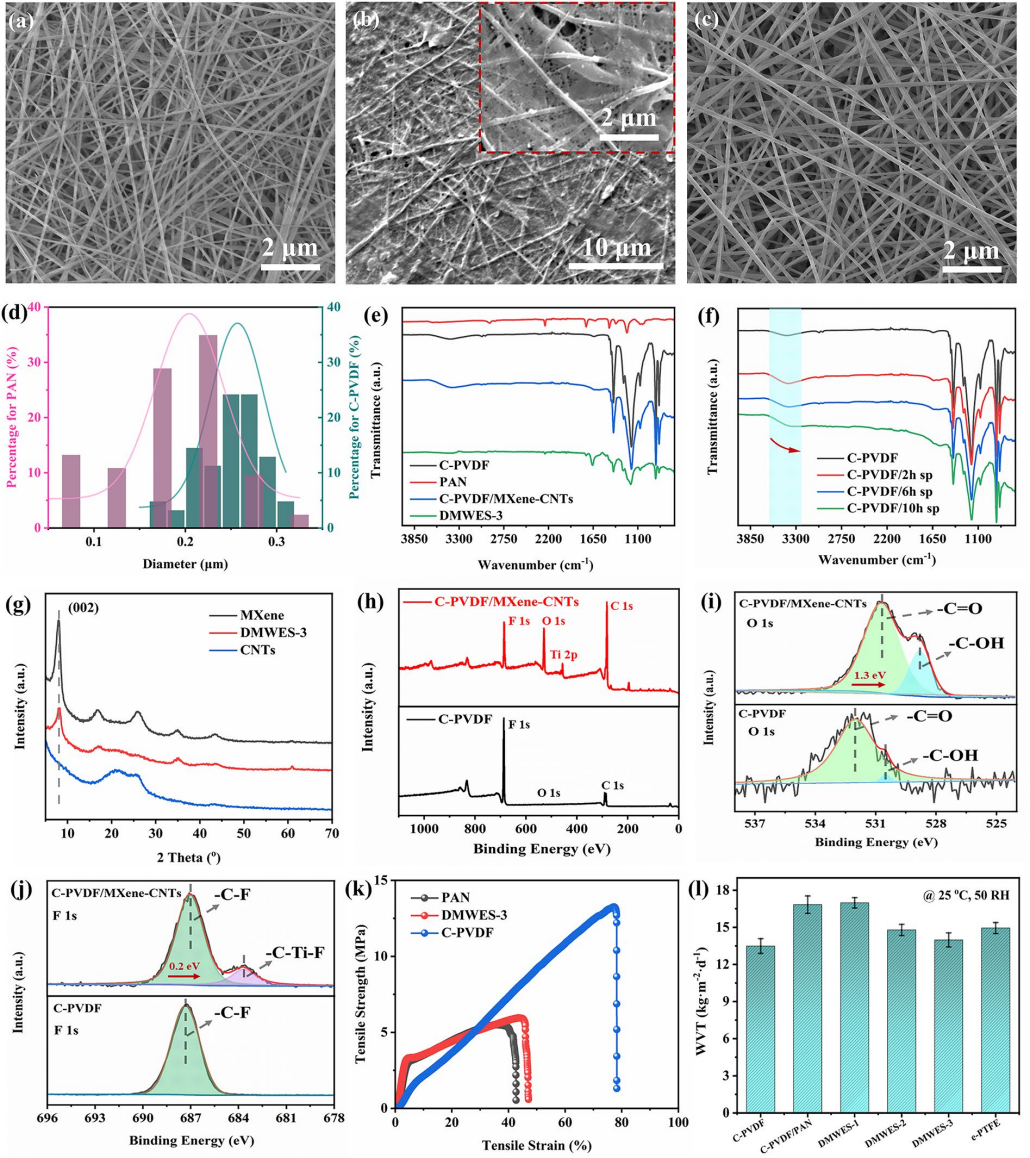
Material characterization of the DMWES.** a** SEM image of C-PVDF nanofibers layer. **b** SEM image of C-PVDF/MXene-CNTs layer, the inset is enlarged image. **c** SEM image of PAN nanofibers layer. **d** Diameter distribution of the C-PVDF nanofibers and PAN nanofibers. **e** FT-IR measurement of the products. **f** FT-IR measurement of the C-PVDF with different time of electrospraying MXene/CNTs. **g** XRD patterns of the products. **h** XPS survey scan of the C-PVDF and C-PVDF/MXene-CNTs. **i, j** O 1*s* and F 1*s* XPS scan spectra of the C-PVDF and C-PVDF/MXene-CNTs. **k** Tensile strength curve of the products. **l** Water vapor transfer rate (WVT) of the products

Figure 2a, c depicts the morphology of the C-PVDF hydrophobic nanofibers layer and the PAN hydrophilic nanofibers layer, respectively. The nanofibers exhibit the randomly distributed nonwoven fabric morphology. Figure 2b shows the morphology of electrosprayed MXene/CNTs ink (DMWES-3) on the C-PVDF substrate; the MXene nanosheets and CNTs were uniformly distributed on the hydrophobic layer and maintained the porous structure of hydrophobic layers at the same time. The SEM images of DMWES-1 and DMWES-2 with different electrospraying time are shown in Fig. S4. The most uniform deposited MXene/CNTs was obtained by DMWES-3 with increased electrospraying time. In addition, the surface resistance changes of the DMWES are shown in Fig. S5. Larger surface resistance of the DMWES-1 and DMWES-2 should be ascribed to the incomplete conductive paths with low MXene/CNTs electrospraying time. The fiber’s average diameters for C-PVDF layer and the PAN layer are about 200 and 260 nm, respectively. C-PVDF layer changes by approximately an order of magnitude, while PAN layer shows more uniform sized fibers (Fig. 2d). Meanwhile, the mean pore diameters generated of PAN layer and C-PVDF layer due to fiber interlaced with each other are 0.47 and 0.507 μm (Fig. S6), which grows by layer and may be conducive to the enhanced capillary force of the hydrophilic pores.

The chemical properties of the DMWES membranes are performed by FT-IR (Fig. 2e). The spectrum shows the characteristic stretching vibration of the nitrile group (C≡N) of PAN at 2,240 cm^−1^. The spectrum of C-PVDF nanofibers displays the typical in-plane bending of -CH2 group at 1,405 cm^−1^ and symmetric stretching of –CF2 group at 1,170 cm^−1^. A wider peak shifting to a lower wavenumber at 3,450 cm^−1^ of -OH group indicates the possible existence of hydrogen bonds between the C-PVDF nanofibers matrix and MXene/CNTs inks (Fig. 2f) [42]. Figure 2g shows the XRD patterns of MXene, CNTs, and DMWES-3; the characteristic peak of Ti_3_C_2_T_x_ at 2*θ* of 6.9° indicates the diffraction from the (002) phase of Ti_3_C_2_T_x_. The XPS survey scan exhibits the coexistence of C, O, F, and Ti elements in C-PVDF/MXene-CNTs (Fig. 2h). Figure 2i, j exhibits the high-resolution O 1*s* and F 1*s* spectrum of C-PVDF and C-PVDF/MXene-CNTs. For O 1*s* spectrum, the peaks at 532 and 530.5 eV are associated with -C = O bond and –C–OH bond of C-PVDF (Fig. 2i), respectively. The peaks are negatively moved to lower binding energy by about 1.3 eV in C-PVDF/MXene-CNTs via the –F…H–O interaction between the –F on C-PVDF and –OH group of carboxylated CNTs [43, 44]. The –C–F peak of C-PVDF from F 1*s* spectrum is shifted to a lower binding energy by ~ 0.2 eV in the electrosprayed sample (Fig. 2j) [45]. The consistent result was also observed from the high-resolution C 1*s* spectrum (Fig. S7). These results demonstrated the formation of stable interface interaction between C-PVDF and MXenes/CNTs. The tensile-strength of nanofibers membrane is of great significance to the wearability evaluation of the fabric based wearable electronics. It can be observed from Fig. 2k that the strength of the DMWES membrane was slightly enhanced to 6 MPa because of the high strength of the hydrophobic C-PVDF layer. Good water vapor transfer rate (WVTR) has a significant impact on the wearing experience. The DMWES membrane with porous gradient structures and asymmetric wettability can continuously transport water vapor from the hydrophobic C-PVDF layer to the hydrophilic fibrous PAN layer rapidly. As a result, the WVT of the DMWES-3 at 25 °C and 50% relative humidity (RH) can attain a WVTR of 13.99 kg m^−2^ d^−1^, which decreases slightly with the increase in the electrospraying MXene/CNTs amount (Fig. 2l). The value is comparable to the WVTR of commercial e-PTFE (15.1 kg m^−2^ d^−1^), indicating good wear comfort.

### Directional Moisture-Wicking Ability of DMWES Membrane

**Fig. 3:**
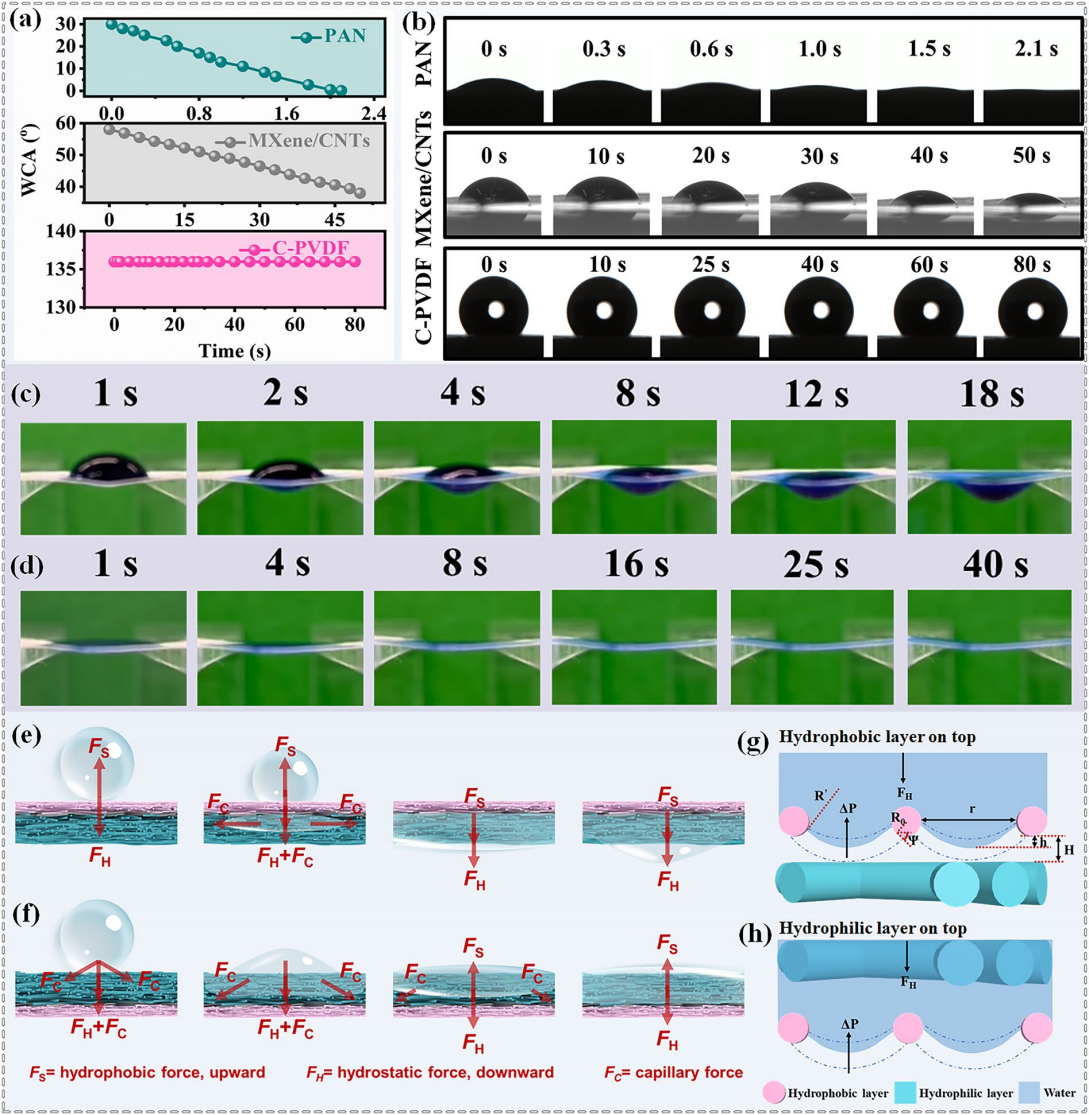
The directional water transport properties of the DMWES. **a, b** Water contact angle change with time increasing. **c** Optical photographs of apparent contact angle change on the hydrophobic C-PVDF nanofibers. **d** Optical photographs of apparent contact angle change on the hydrophilic PAN nanofibers. **e, f** Proposed directional water transport mechanism of the DMWES form the hydrophobic and hydrophilic layer. **g, h** Proposed analysis model of the implementation of the directional water transfer in two situations

In order to evaluate the directional water transport performance in the DMWES membrane, we studied the wettability of the hydrophilic and hydrophobic nanofiber membranes firstly. Figure S9 exhibits the cross-sectional image of the DMWES membrane with the thickness of about 15 and 60 μm for the hydrophobic C-PVDF nanofibers layer and superhydrophilic PAN nanofibers layer, respectively. Figure 3a, b shows the water contact angle (WCA) change of each single layer with time increasing. On the PAN nanofibrous layer, the WCA dynamically decreases from ~ 31° initially to 0° within 2.1 s, showing the superhydrophilicity of the nanofibrous PAN. The C-PVDF layer indicates the stable hydrophobic behavior. The water droplet with an initial WCA of 138° and no obvious spreading on the hydrophobic C-PVDF nanofibers are observed. The water transport capability of the DMWES membrane was monitored by the dynamic transfer process of water droplet on both sides of DMWES membrane. When a water drop contacted the hydrophobic C-PVDF layer, the water was pumped over the hydrophobic side and wetted the superhydrophilic PAN nanofibers side in about 18 s (Fig. 3c). In contrast, when the DMWES membrane was flipped and the water droplet contacted the superhydrophilic nanofibrous PAN layer, the water droplet was blocked and spread over the superhydrophilic nanofibers without penetrating the hydrophobic C-PVDF nanofibers (Fig. 3d). These results suggest that the DMWES membrane can transport liquid unidirectionally from the hydrophobic side to the superhydrophilic side (Movie S1). We here regulated the thickness of the C-PVDF nanofibers layer to assess the thickness effect on directional water transport performance. When the thickness of the C-PVDF increased to 18 μm, the water droplet transferred from the upper hydrophobic layer to the downward hydrophilic layer in 25 s because of great hydrophobic force (Fig. S10). When the thickness of the C-PVDF increased to 30 μm, the water droplet cannot be transferred from the upper layer to the downward layer (Fig. S10). In the reverse direction, the water droplet cannot penetrate into the downward hydrophobic nanofibers layer in these two conditions, and it can just spread on the hydrophilic nanofibers layer.

We proposed a possible mechanism for both conditions from the macroscopic force analysis in Fig. 3e, f. When the hydrophobic C-PVDF nanofibers layer is facing upward and the droplet touches the surface, the droplet will be subjected to the upward hydrophobic force (F_S_) and the downward hydrostatic pressure (F_H_). The F_H_ is directly proportional to the height and mass of the droplet. As the droplet increases in size and time, the F_H_ will break through the water transfer barrier force because of the F_S_ of the C-PVDF nanofibers. The water will be trapped by the hydrophobic pores and come to contact the underlying superhydrophilic nanofibers. After that, the droplet will be exposed to strong capillary force (F_C_*,* Eq. 1) in all directions except upward, which is generated by the Laplace pressure.

1 $$\mathrm{F_{C}} =\frac{2\gamma \mathrm{ cos} \theta }{r}$$

where *r*, *γ*, and *θ* are the pore radius, the liquid surface tension, and the liquid contact angle, respectively. Obviously, F_C_ is inversely proportional to the pore size, demonstrating that the gradient porous structure plays an important role in the water transport process. The decreased pore size of the PAN nanofibers resulted in the increase in the F_C_ in both the vertical and the horizontal directions. The F_C_ will continually pull water into the internal superhydrophilic nanofibers layer through numerous pores of the C-PVDF nanofibers. And then the F_S_ will reverse and further facilitate the downward transfer of water droplets. Finally, the liquid will drip from the superhydrophilic PAN nanofibers (Fig. 3e). When the DMWES is reversed, the droplet will be subjected to the strong capillary force F_C_ of the PAN nanofibers from every direction except upward until the superhydrophilic nanofibers are fully wetted. The lower hydrophobic C-PVDF nanofibers substrate will then exert an upward F_S_ on the contacted liquid to prevent the liquid from dripping (Fig. 3f) [46, 47].

The model of the superhydrophilic and hydrophobic nanofibers was further simplified in microscopic scale to understand the key parameters of directional water transport. As shown in Fig. 3g, when the hydrophobic nanofibers face up, the liquid-solid-gas three-phase contact line (TCL) will go lower toward an equilibrium position after dipping water and form the convex surface between the hydrophobic fibers as the initiating surface. The convex surface is then subjected to an upward force (Δ*P*) calculated by the following Eq. 2 [46]:

2 $$\Delta P =\frac{\upgamma }{{R}^{^{\prime}}}=\frac{2\mathrm{\gamma sin}[\theta \left(\psi \right)-\psi ]}{r+2{R}_{0}(1-\mathrm{sin}\psi )}$$

where r is the pore diameter between the hydrophobic fibers, ψ is the local geometrical angle, and *R*_0_ is the fiber radius. Δ*P* corresponds to the value of F_H_ when TCL reaches an equilibrium state. The TCL goes lower with the increase in F_H_, and the vertical distance h between the bottom of the hydrophobic nanofibers and the convex apex increases. The distance between hydrophobic and superhydrophilic nanofibers is H. To achieve directional water transfer, *h* = H must be satisfied so that the liquid can reach the superhydrophilic fibers. Then, the reverse hydrophobic force (*F*_S_) and strong F_C_ of hydrophilic layer can induce the push–pull effect to drag the liquid to the pores of the hydrophilic nanofibers. Nevertheless, when the thickness of hydrophobic nanofibers increases, Δ*P* will increase because of the decreased *r* value by mutual stacking of nanofibers; hence, *h* will decrease accordingly. The *h* will be much smaller than *H* in this case, so directional water transport cannot be implemented. When the hydrophilic layer is on top (Fig. 3h), water droplets will not drip down from under the hydrophobic fibers, because the air can be regarded as absolutely superhydrophobic and *h* is infinitely less than* H*. Herein, the directional moisture-wicking multilayered nanofibers membrane should meet the following four requirements: (1) The hydrophobic nanofibers should be hydrophobic enough to provide adequate *F*_S_. (2) The hydrophobic fibers have to be thin to allow water to go through quickly. (3) The hydrophobic layer should have numerous pores, and the gradient pore size from the hydrophobic layer to the hydrophilic layer will induce higher capillary pressure as the driving force to extract the water efficiently. (4) The hydrophilic layer should be relatively thick and have excellent hydrophilicity to directly extract the moisture.

### Pressure Sensing Performance of DMWES Membrane

**Fig. 4:**
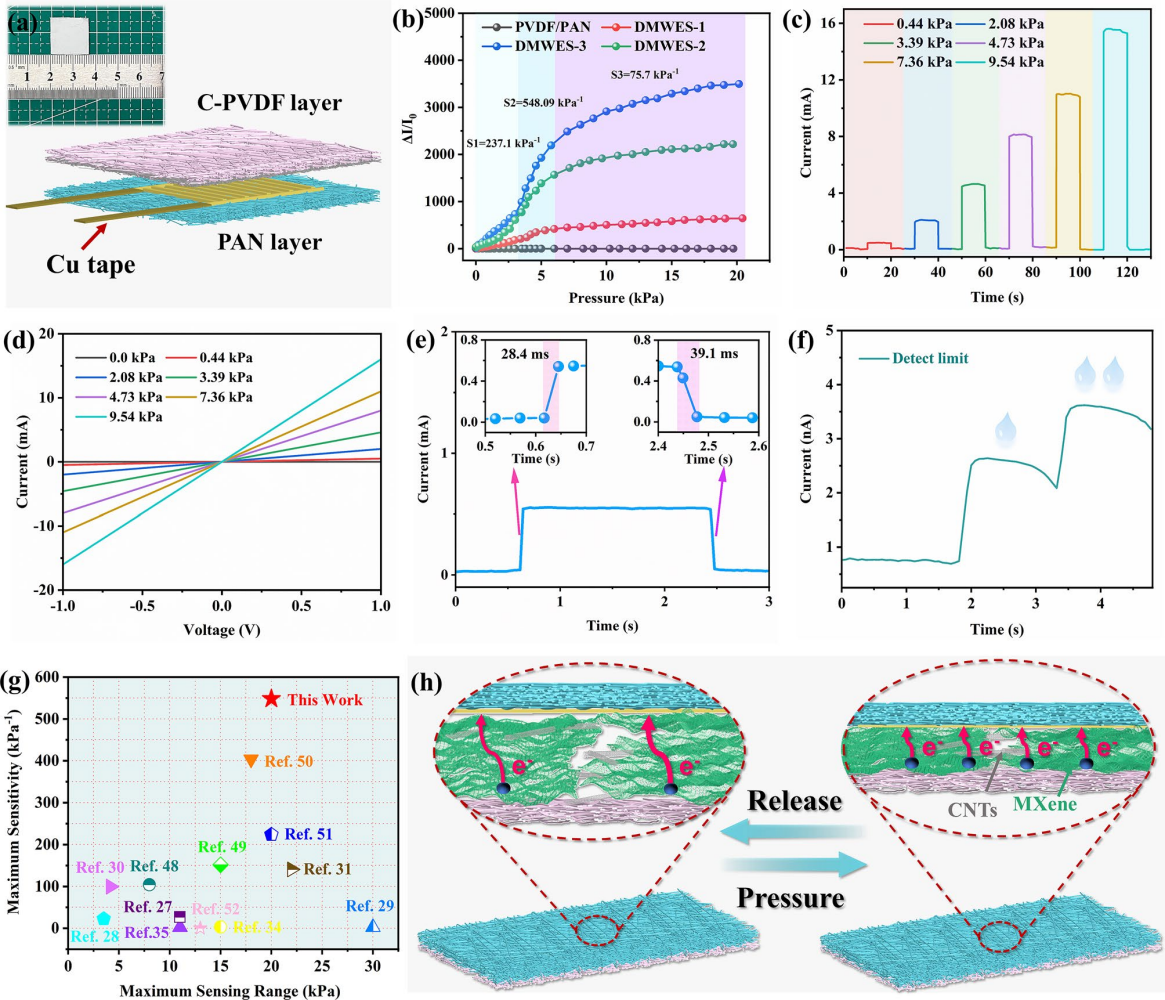
Pressure sensing performance and the sensing mechanism of the DMWES. **a** Schematic illustration of the DMWES as pressure sensors. **b** Sensitivity curve of the DMWES with different electrospraying time. **c** Current response curves of the DMWES under serial pressures. **d** *I-V* curves of the DMWES under serial pressures. **e** Response and recovery time of the DMWES. **f** Detection limit of the DMWES. **g** Comparison of the maximum sensitivity and maximum sensing range with other reported studies. **h** Schematic illustration of the sensing mechanism of the DMWES

To test the sensing performance of the DMWES membrane, we cooperatively utilized the piezoresistive effect and the triboelectric effect of the fabricated electronic skin to realize all-range healthcare monitoring, and biomechanical energy harvesting. Firstly, the electromechanical properties of DMWES are measured by monitoring the relative resistance variation or sensitivity (Δ*I*/*I*_0_, Δ*I* = *I − I*_0_, *I*_0_ indicates the original current, *I* represents the current under pressure). Figure 4a shows the schematic diagram of the sensing elements, and the inset is the picture of real product with the size of 1.6 × 1.6 cm^2^. An interdigital Au electrode was sputtered on the PAN nanofibrous membrane to contact the MXene/CNTs sensing layer and collect the electric signal (Fig. S11). Figure 4b shows the sensitivity curve of the DMWES with different MXene/CNT contents under wide range of pressure. The DMWES-3 is observed to have the highest sensitivity value in comparison with the others, which can be ascribed to the uniformly distributed MXene/CNTs sensing layer. The DMWES-1 and DMWES-2 show lower sensitivity in comparison with DMWES-3, which can be attributed to the uneven spraying of MXene/CNTs on the C-PVDF nanofibers matrix and thus, larger interface resistance and poor ohmic contact between the conductive sensing layer and interdigital electrode, resulting in low sensitivity. The sensitivity curve can be split into three parts: S1 exists in the low-pressure area (0–3.20 kPa), S2 lies in the middle-pressure area (3.20–6.30 kPa), and S3 lies in the high-pressure area (6.30–20.0 kPa). The corresponding sensitivity of S1, S2, and S3 is 237.1, 548.09, and 75.7 kPa^−1^, respectively. In the low-pressure range, the current response and sensitivity of the DMWES are relatively lower than the sensitivity at the medium-pressure range, which is due to the small air gap between the interdigital electrode and the conductive MXene/CNTs layer and the energy absorption effect of the nanofibers. The following measurements of other performance were herein determined by the DMWES-3. The current–time (*I-T*) curves show a gradual increasing trend in the current under the correspondingly increased external pressure (Fig. 4c). Figure 4d exhibits the current–voltage (*I-V*) curves of the DMWES-3 with the voltage sweeping from − 1.0 to 1.0 V, showing the proportional increasing dependence of the voltage by the current under different pressure. This finding demonstrates a good ohmic contact between the conductive sensing layer and the interdigital Au electrode, enabling the DMWES membrane to distinguish different pressures over a wide range. Furthermore, the electronic skin displays a fast response (28.4 ms) and recovery time (39.1 ms) because of the superior contact resilience of the C-PVDF nanofibers film and interdigital electrode (Fig. 4e). The lowest detection limit of the DMWES is one of the critical factors in determining the scope of electronic skin applications. Here, two drops of water were utilized to evaluate the detection capability of the DMWES at extremely low pressures. As shown in Fig. 4f, the slight force imposed by a drop of water (~ 5 Pa) can be exactly detected by the DMWES membrane. By comparing the maximum sensing range and sensitivity with the previous studies, DMWES in this work exhibits comprehensive advantages (Fig. 4g), which would show great potential in various application scenarios. The long-term piezoresistive stability of the DMWES was further evaluated by continuous compression and separation test at 10 kPa; the current response under pressure was stable even after 3,000 cycles (Fig. S12). The comprehensive performance comparison with more parameters, including the response/recovery time and detection limit, is displayed in Table S1 [48–52]. The proposed sensing mechanism of the DMWES is shown in Fig. 4h. The DMWES is equivalent to a serial circuit that contains three variable resistances. The equivalent circuit of the resistance is exhibited in Fig. S13. When the external pressure is imposed on the electronic skin, the contact point in the conductive network of C-PVDF/MXene-CNTs increases relatively. A significant decrease in the contact resistance (*R*_i_) and bulk resistance (*R*_b_) of the DMWES membrane will be observed. The *R*_i_ change of the DMWES is determined by the contact dimension between the MXene-CNTs electrospraying side and interdigital electrode. The sensing layer and the electrode are not in close contact with each other in the original no-pressure condition; hence, a high *R*_i_ exists initially at their interface, and then, it decreases under increased pressure. *R*_i_ exhibits a significant response to tiny pressure. When the DMWES is exposed to higher pressure, the *R*_i_ variation will attain saturation rapidly, and *R*_b_ will decrease gradually under higher pressure, giving the DMWES a wider detection range.

### Triboelectric Nanogenerator Performance of DMWES Membrane

**Fig. 5:**
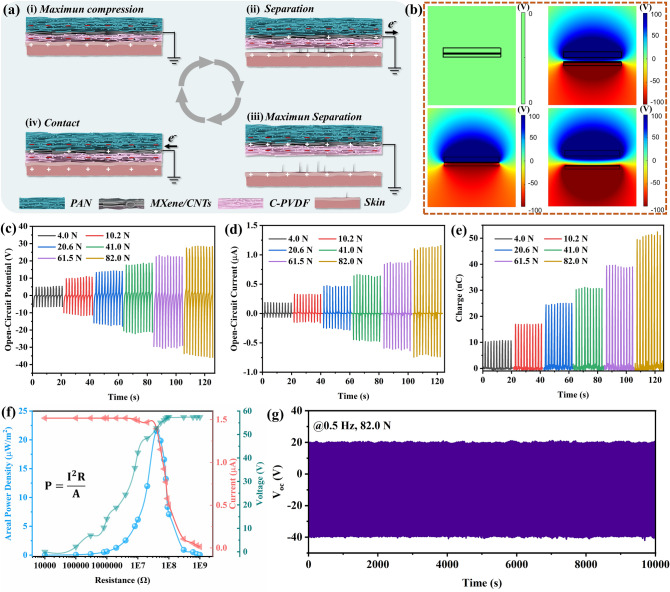
Performance of the DMWES as STENG. **a** Schematic working principle of the DMWES under triboelectric sensing. **b** Numerical calculations of the potential distribution of the DMWES at the contact and separation states. **c**, **d**, **e** *V*_OC_, *I*_SC_, and *Q*_SC_ of the TENG based on the DMWES under different forces. **f** Voltage and current, and areal power density of the DMWES at various external resistance loadings. **g** Cycling performance of continuous 5,000 cycles working of the DMWES

On account of the high conductivity of MXene/CNTs electrospraying layer and strong electronegativity of the MXene/CNTs, C-PVDF, and PAN layers, DMWES was further explored based on the single-electrode triboelectric mechanism for biomechanical energy harvesting, in which the MXene/CNTs electrospraying side acted as the electrode. Figure 5a demonstrates that the DMWES in single-electrode pattern comprises of two sections: MXene/CNTs electrospraying side as electrode layer, DMWES and human skin as negative triboelectric and positive layers, respectively. When the skin (or other counterparts) is in contact with the DMWES, the skin will transfer the charges to DMWES due to the high surface electron affinity of the DMWES membrane. Once the separation occurs, the potential difference is generated. Hence, the DMWES continuously produces an alternate electricity (AC) signal through the entire cycle of contact and separation processes. Figure 5b presents the simulated electric potential distribution by COMSOL multiphysics, explaining the electricity generation process of during the contact and separation process of triboelectrification quantitatively. With the maximum degree of contact and separation, the simulation output of the single-electrode triboelectric nanogenerator (STENG) reaches the maximum value of about 200 V. Figure 5c-e demonstrates the triboelectric output of DMWES when pairing with aluminum foil as the positive counterpart, the output performance shows the linearly proportional force-dependent relationship until 82.0 N, which can generate the maximum output including the *V*_OC_ = 62 V, *I*_SC_ = 1.6 μA, and *Q*_SC_ = 49 nC at the force of 82.0 N, respectively. We also conducted water spraying treatment for the TENG to simulate the influence of water and sweating on the output performance. When spraying water on the TENG during the test, the TENG output decreased rapidly and then, gradually recovered after a certain period of self-drying, which can be restored to about 80% of the original state in 10 min (Fig. S14). Figure 5f shows the output voltage, current and areal power density of the DMWES membrane with external resistance loadings from 10 kΩ to 1 GΩ. The areal peak power density of the DMWES was calculated from the equation of *P* = *I*^2^*R*/*A*, where *A*, *R*, *I*, and *P* represent the contact area, resistance loading, output current, and power density of the DMWES, respectively. The areal peak power density of the DMWES can reach a peak value of 21.6 µW m^−2^ at the external resistance loading of 40 MΩ. Furthermore, the durability of the DMWES is demonstrated in Fig. 5g. The generated V_oc_ nearly appeared as a constant of ~ 60.5 V after 5,000 continuous cycles at a frequency of 0.5 Hz and 82.0 N loading force, showing excellent mechanical robustness and stability.

### All-Range Healthcare Sensing of DMWES Membrane

**Fig. 6:**
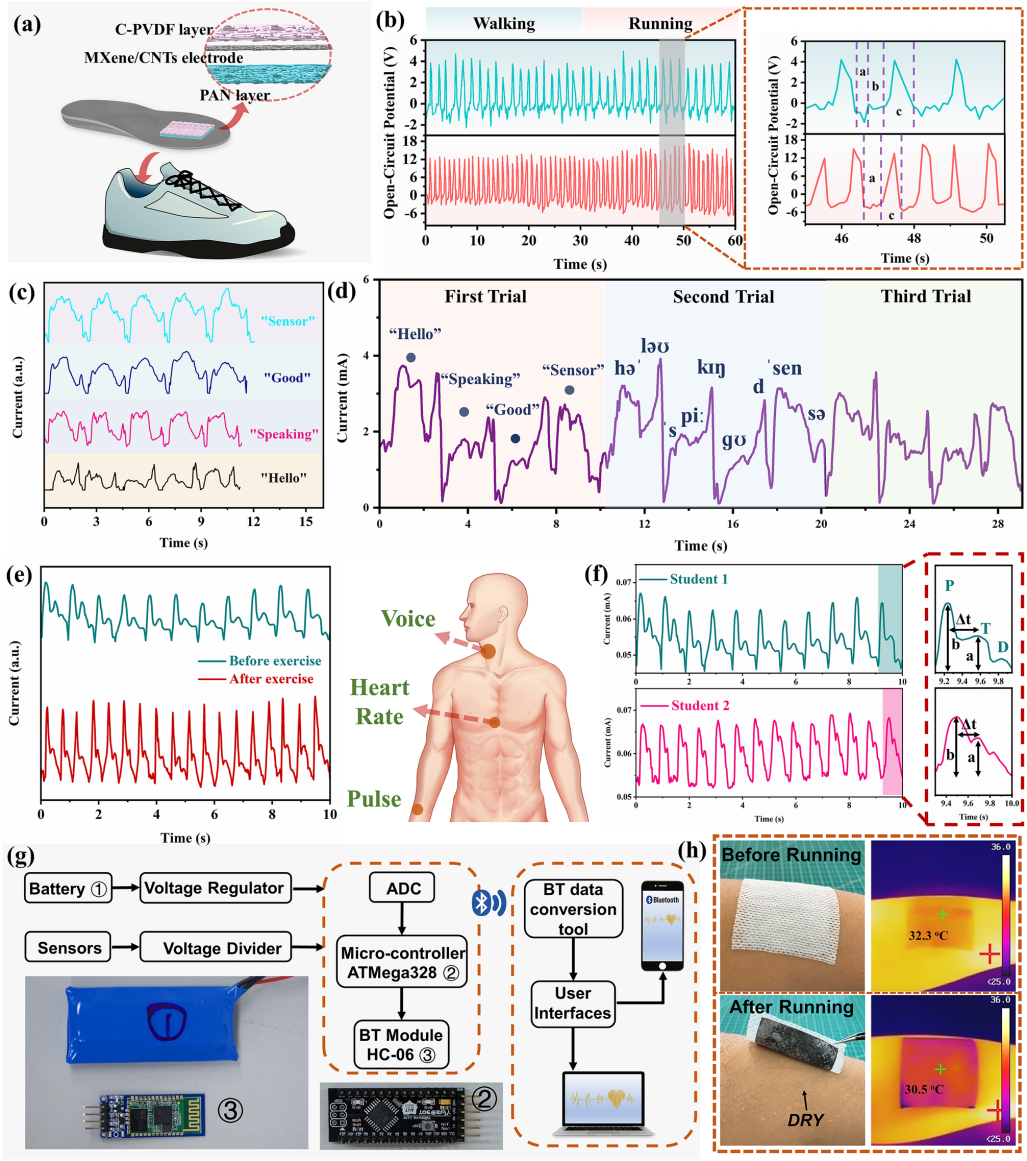
DMWES for all-range health monitoring. **a** Gait sensing scheme of the DMWES. **b** The gait sensing signal and enlarged signal of the DMWES. **c** Voice signal of the different words. **d** Voice signal of the sentence of continuous words speaking. **e** Wrist pulse signal of a 28-year-old male man before and after exercise. **f** Wrist pulse signal of two students. **g** Schematic illustration of the signal acquisition and analysis system including signal acquisition, processing, wireless transmission, and mobile application. **h** Optical and Infrared camera images of the DMWES on skin before and after running exercise

Based on the superior pressure sensing and triboelectric performance of the DMWES membrane, the DMWES can realize the all-over healthcare sensing with these two mechanisms. In Fig. 6a**,** the DMWES membrane was used to gather gait information and biomechanical energy based on the single-electrode triboelectric sensing mechanism during running exercise, which was simply pasted onto the internal sole of the running shoe. Distinct signal patterns generated when the volunteer was walking at different paces, which could be acquired by analyzing the frequency and amplitude of the obtained signals easily (Fig. 6b). The *V*_oc_ signal of running (1 Hz frequency) was approximately two times higher than the generated signal of walking (0.5 Hz frequency). Additionally, the walking pace can be resolved into three sections from the one-step signal in Fig. 6b (*i.e.*, a. stomping, b. intermission, c. lifting and striding). Two inverse *V*_oc_ peaks could be observed in stage a and stage c, indicating the imposed pressure by feet during walking. The intermission could be applied to demonstrate the walking pace. The stage b disappeared when the feet frequency reached 1 Hz, indicating fast running. The walking information is of great value to physicians and coaches in the physical therapy of patients with lower extremity disorders and scheduling training sessions for athletes. In addition, the DMWES can achieve the accurate physiological monitoring of weak bio-signal based on the piezoresistive pressure sensing. The DMWES exhibited clear and steady signals of various degree of joint motions (Fig. S15a) and displayed a high sensitivity for articular nimbleness monitoring. Figure S15b shows the 7 cycles of breathing signal in 18 s, which is consistent with the normal adult breathing frequency of 16–24 cycles in one minute. Figure 6c shows the DMWES attached onto the volunteer’s throat to monitor the tiny movement of vocalization. The DMWES produces apparent signal patterns for different word vocalization, *i.e.*, “hello” “speaking” “good,” and “sensor,” demonstrating the potential application in AI and voice recognition. Figure 6d shows the signal record of words spoken together in three trials, indicating the consistency of the voice signal. Interestingly, the experimental signal results represent the similar characteristic peaks existing in corresponding tones of word signals. Pulse waves provide vital information on heart conditions and related diseases. Generally, a radial artery wave could be observed with the characteristic percussion, tidal, and diastolic peaks (P-wave, T-wave, and D-wave, respectively) [53]. Further, we applied this DMWES to monitor human pulse waves by attaching the DMWES to the wrist (Fig. 6e, f). Figure 6e shows the pulse wave of a 28-year-old male man before and after work-out. The pulse rate and amplitude both increased after a 10 min rope skipping exercise, and the waveform changed as well. Specifically, the pulse rate increased from 66 beats per minute (BPM) in the resting state to 104 BPM in the exercising state. The tidal peak came to be blurred and the relative strength of the diastolic peak to the percussion peak reduced on the waveform. It was worth noting that the all-fiber based DMWES exhibited stable pulse signal even after exercise, demonstrating that the directional moisture transport capability enabled the relatively stable test environment of MXene/CNTs sensing layer. We also evaluated the pulse monitoring performance of the DMWES in the simulated sweat environment with NaCl solution (Fig. S16). It was observed that the DMWES could still clearly detect pulse signals in the simulated sweat. But the current intensity was higher because of more conductive paths as water penetrated into the conductive and hydrophilic layers. And the increased internal contact interfaces resulted in the decrease in the pulse peak intensity variation (Movie S2). In Fig. 6f, we tested the pulse wave of two students (a 28-year-old male man and a 30-year-old male man for student 1 and student 2, respectively). The analysis of stationary signals shows radial artery frequencies of ~ 66 and ~ 84 BPM, respectively, indicating the good health state of two students. As presented in the enlarged pulse of Fig. 6f, the typical peaks (P-wave, T-wave, and D-wave) were clearly captured and distinguished. We can also observe significant health indicators like reflection index (*R.I.,* Eq. 3) and stiffness index (*S.I.,* Eq. 4) from the response time by resolving the two distinct peaks of P-wave and T-wave (Δ*t*), and the intensity of P-wave and T-wave (b and a, respectively) [54]. The stiffness of the vessels was assessed by the measurement of *R.I.* and *S.I.*:

3 $$R.I. =\frac{a}{b}\times 100\%$$

4 $$S.I. =\frac{\mathrm{Subject\; height}}{\mathrm{\Delta t}}\; (\mathrm{m}/\mathrm{s})$$

The *R.I.* and *S.I.* values were computed to be 50.1% and 5.40, 64.3% and 9.38 for student 1 and student 2, respectively. The results indicated the healthy status of the two students. The higher the *R.I.* and *S.I.* values, the higher the risk of cardiovascular disease like hypertension, cardiovascular infarction.

We further developed a wearable physiological monitoring system connecting the DMWES for ECG detection. As shown in Fig. 6g, the main components of the system are exhibited, including data acquisition and processing unit, wireless data transmission module, etc. We designed a circuit that used a frequency divider circuit structure to transform the resistance of the DMWES into a voltage, which was then converted to the digital domain by an analog-to-digital converter (ADC). The analog output of the driver circuit was synchronously digitized using a high-precision ADC chip. The digital signals were processed by a microcontroller (ATMega328) and then, transmitted wirelessly at 100 Hz via a Bluetooth module (HC-06). A Bluetooth receiver was used to convert the digital signals from the DMWES into analyzable data for smartphones. Our DMWES was led out from both ends of the interdigital electrode through copper tapes and then, connected to the PCB. The student 1’s ECG signal was received by putting the DMWES membrane on the chest (Movie S3). The generated ECG pulse signal was extracted from the pulse system and observed for ~ 71 BPM, which proved the good healthy state of the student 1 (Fig. S17). These health parameters are of great significance in cardiovascular disease monitoring [55, 56]. In the end, we compared the moisture-wicking effect of our product and the commercial product after running exercise. Figure 6h shows the optical and infrared sweating pictures of the DMWES membrane before and after running exercise. When the DMWES membrane was lifted up, we could see that the skin beneath it was still dry. And the infrared picture demonstrated that the surface temperature of the DMWES membrane decreased about 1.8 °C after running because of the transferred moisture from the bottom hydrophobic layer, assuring the user’s comfort and fast drying of inner layer, and signal stability. However, there was a lot of perspiration at the contact area between the commercial gel electrode and the skin, and it even fell off from the skin (Fig. S18 and Movie S4). The fabricated DMWES membrane shows great application prospect in wearable electronic textiles with comfortable exercise experience.

## Conclusions

In summary, we have successfully designed an ultrasensitive directional moisture-wicking electronic skin with dual-mode sensing capability and biomechanical energy harvesting based on the construction of heterogeneous fibrous membranes and the controllable MXene/CNTs electrospraying layer. Unidirectional moisture transfer was successfully realized by surface energy gradient and push–pull effect induced by the distinct hydrophobic-hydrophilic difference, which can spontaneously absorb sweat from the skin and ensure bioelectrical signal stability. The directional moisture-wicking electronic skin showed superior static sensing properties, high sensitivity in low-pressure area, wide linear range, rapid response/recover time. In addition, the STENG based on the DMWES can deliver a high areal power density of 21.6 µW m^−2^ and good cycling stability. With the superior pressure sensing and triboelectric performance, the DMWES can achieve all-range healthcare sensing, including accurate pulse monitoring, voice recognition, and gesture sensing. This work helps to lay the foundation for the development for the next-generation breathable self-powered electronic skins in the applications of AI, human–machine interaction, and soft robots.

### Supplementary Information

Below is the link to the electronic supplementary material.

Supplementary file1 (MP4 8721 KB)

Supplementary file2 (MP4 10634 KB)

Supplementary file3 (MP4 1765 KB)

Supplementary file4 (MP4 6446 KB)

Supplementary file5 (PDF 1512 KB)
