# Supplementary material for: Bioinspired All-Fibrous Directional Moisture-Wicking Electronic Skins for Biomechanical Energy Harvesting and All-Range Health Sensing
Source: Nanomicro Lett. 2023 Mar 2;15:60. doi: 10.1007/s40820-023-01028-2 (PMC9981859; doi:10.1007/s40820-023-01028-2)
Supplement: Supplementary file 5 — Supplementary file5 (PDF 1512 KB) [file 40820_2023_1028_MOESM5_ESM.pdf]

Supporting Information for

Bioinspired All-Fibrous Directional Moisture Wicking Electronic Skins for Biomechanical Energy Harvesting and All-Range Health Sensing

Chuanwei Zhi[1], Shuo Shi[1], Shuai Zhang[1], Yifan Si[1], Jieqiong Yang[1], Shuo Meng[1], Bin Fei[2], Jinlian Hu[1, 3, *]

[1]Department of Biomedical Engineering, City University of Hong Kong, 999077, Hong Kong S.A.R, China

[2]Institute of Textiles and Clothing, The Hong Kong Polytechnic University, 999077, Hong Kong S.A.R, China

[3]City University of Hong Kong Shenzhen Research Institute, 518057, Shenzhen, P. R. China

*Corresponding author. E-mail: jinliahu@cityu.edu.hk (Jinlian Hu)

Supplementary Figures and Table

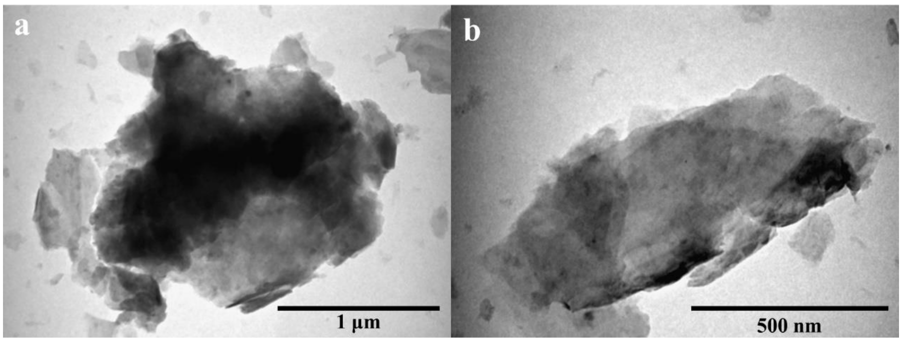

Fig. S1 TEM image of MXene

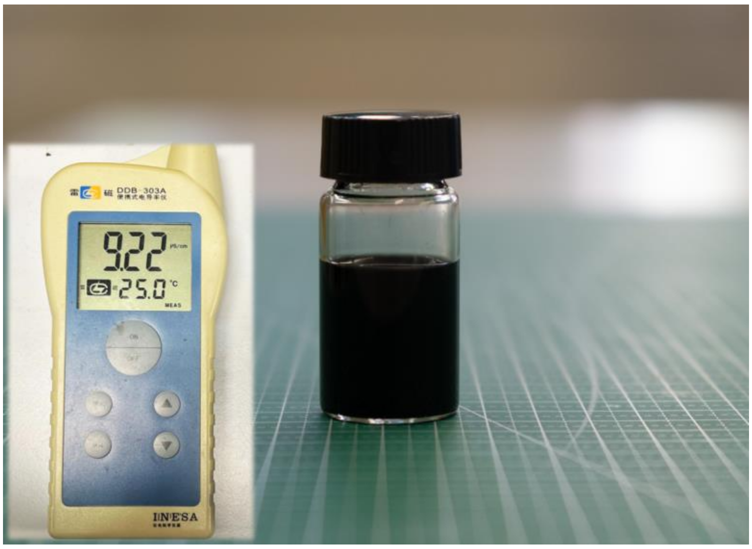

Fig. S2 Optical image and solution conductivity of the MXene/CNTs ink

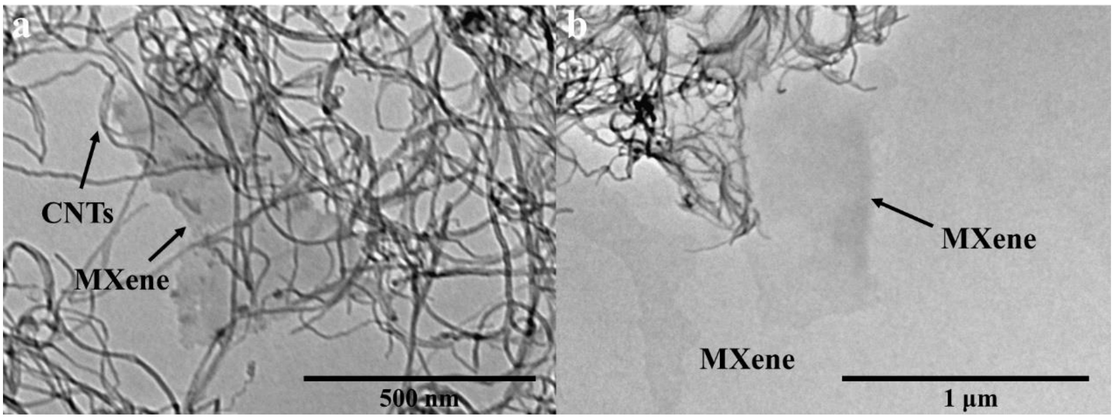

Fig. S3 TEM image of the diluted MXene/CNTs ink

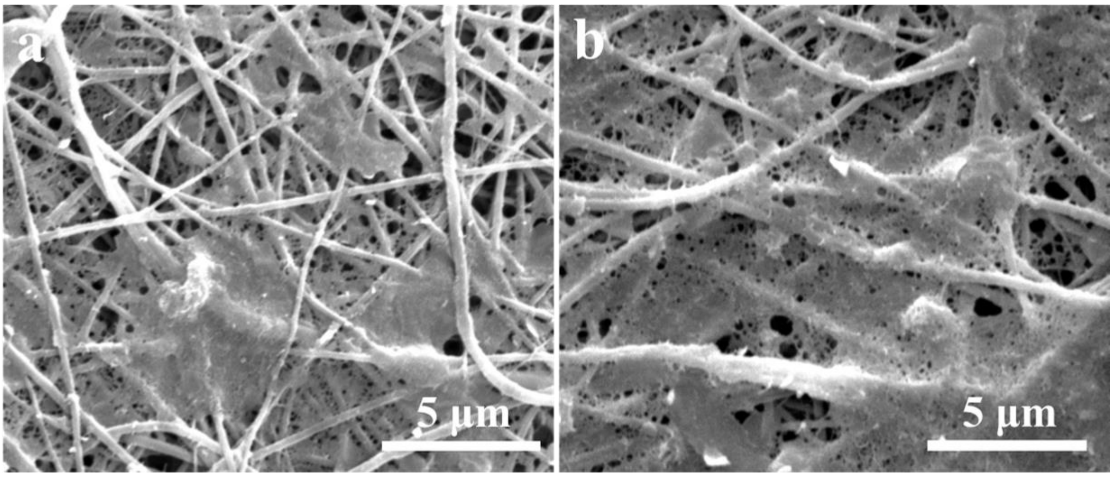

Fig. S4 SEM image of the C-PVDF/MXene-CNTs with different electrospraying time

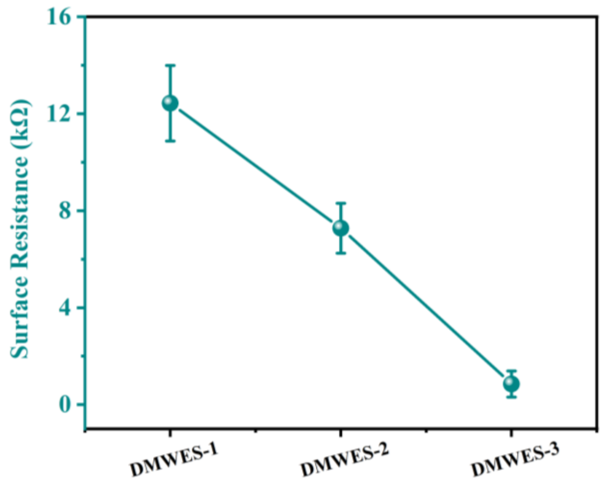

Fig. S5 Surface resistance of the C-PVDF/MXene-CNTs with different electrospraying time

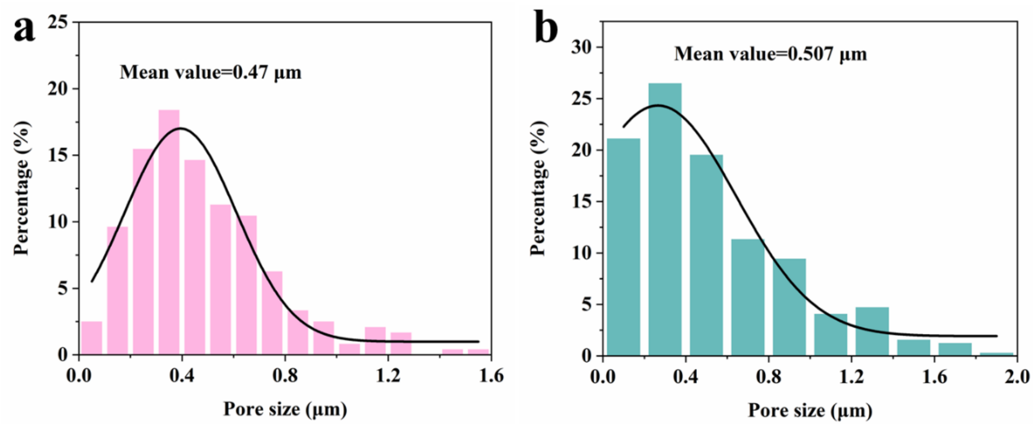

Fig. S6 Pore size distribution of the PAN and C-PVDF nanofibers.

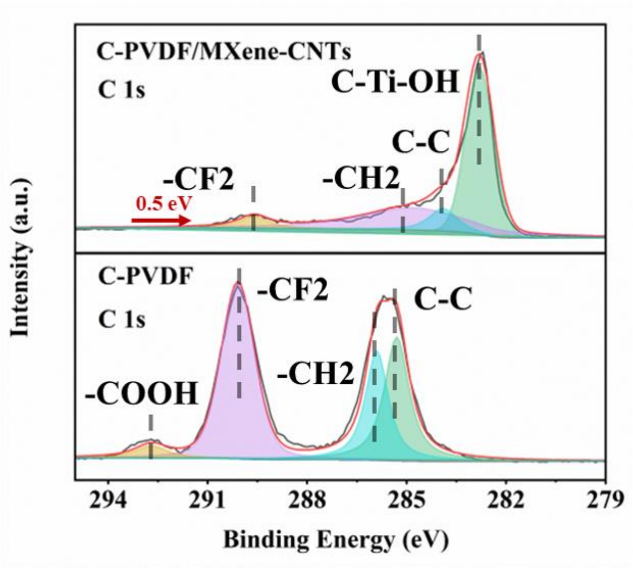

Fig. S7 High-resolution C 1s spectrum of the C-PVDF/MXene-CNTs and C-PVDF

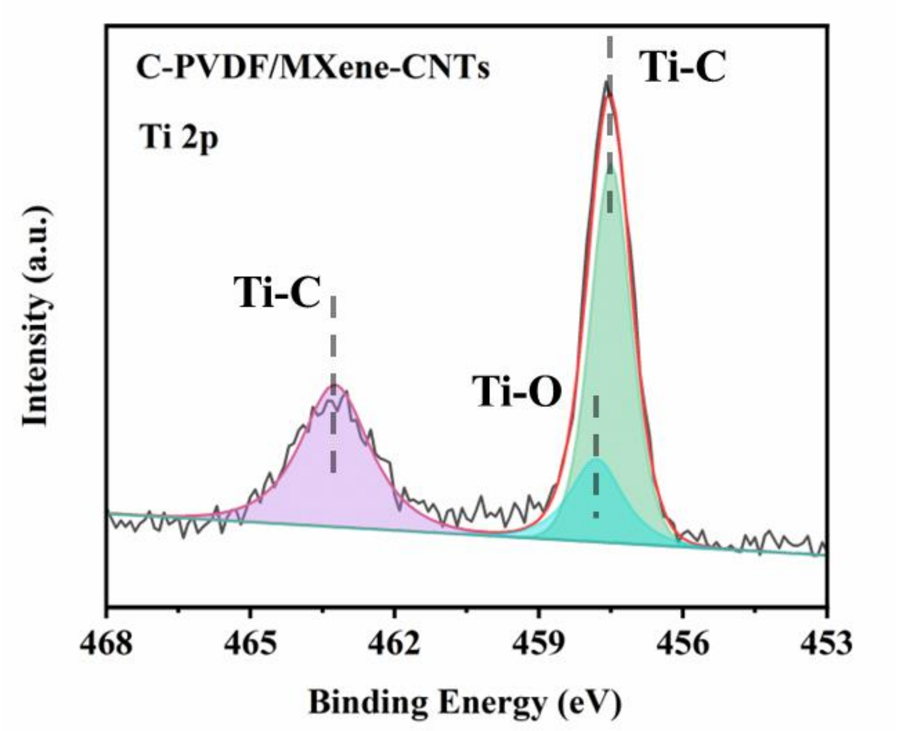

Fig. S8 High-resolution Ti 2p spectrum of the C-PVDF/MXene-CNTs

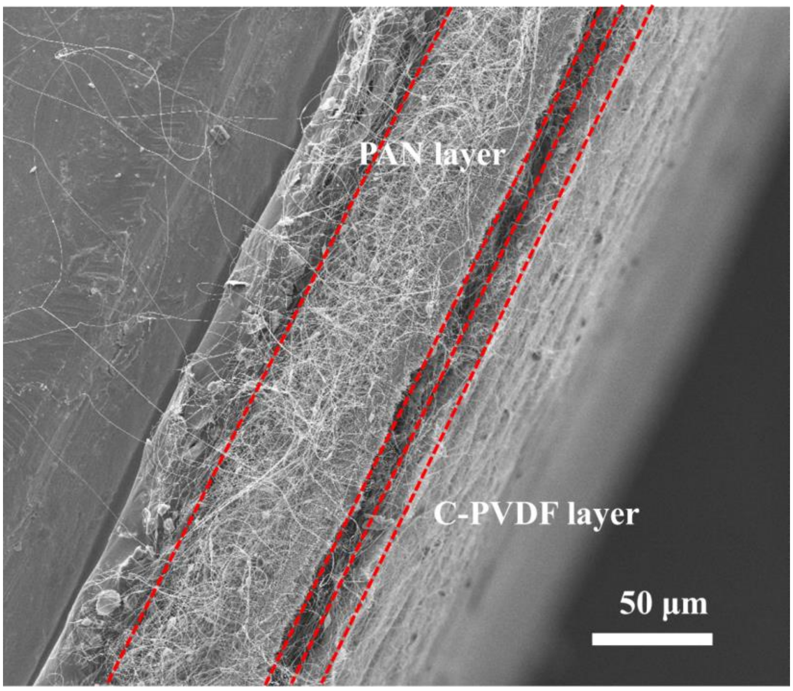

Fig. S9 Cross-sectional SEM image of the DMWES

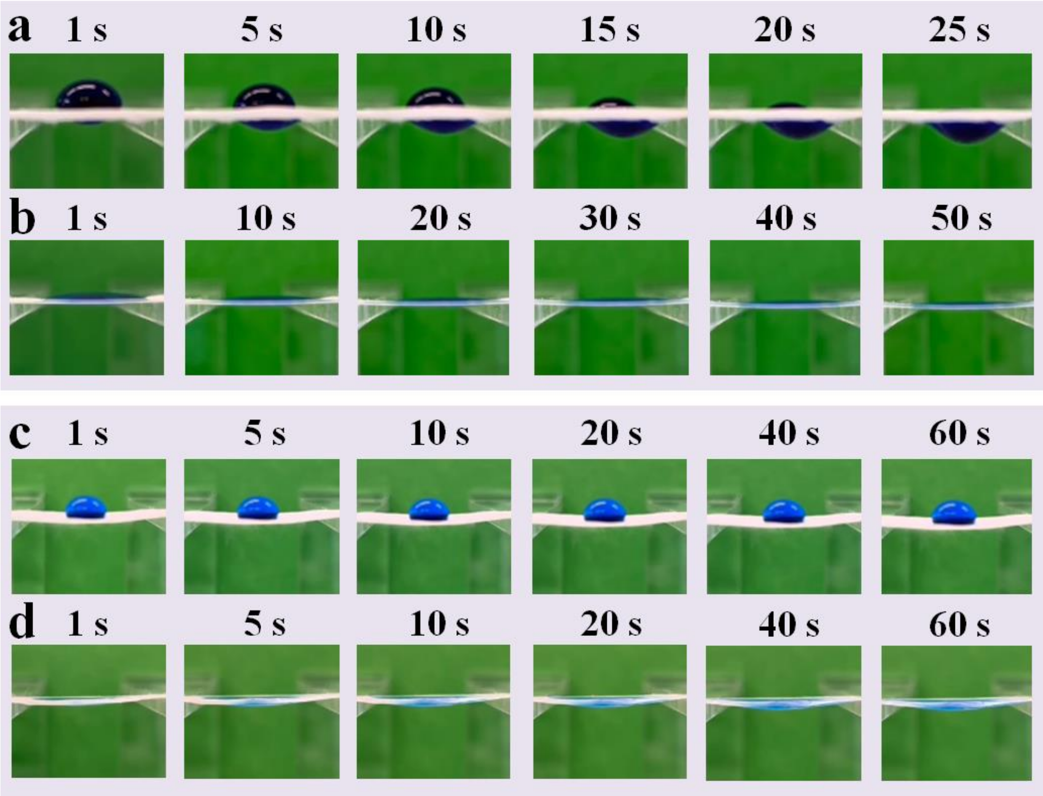

Fig. S10 The optical image of the dynamic water transport of the DMWES with different thickness of C-PVDF nanofibers. (**a, b**) Optical photos of dynamic contact angle change on the hydrophobic C-PVDF nanofibers and hydrophilic PAN nanofibers, respectively, when C-PVDF layer reaches 18 μm. (**c, d**) Optical photos of dynamic contact angle change on the hydrophobic C-PVDF nanofibers and hydrophilic PAN nanofibers, respectively, when C-PVDF layer reaches 30 μm

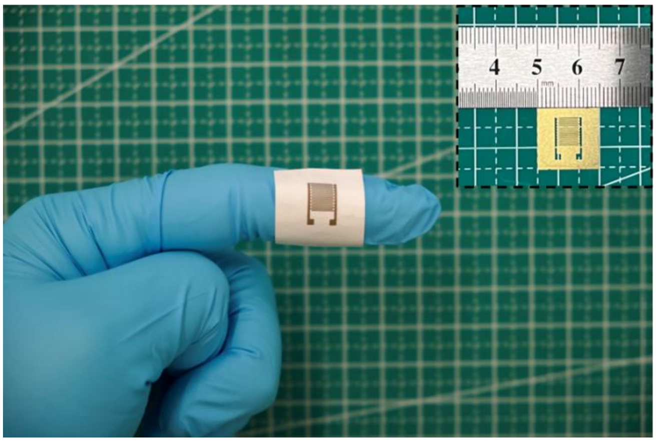

Fig. S11 Optical image of the sputtered interdigital electrode, the inset is the electrode template

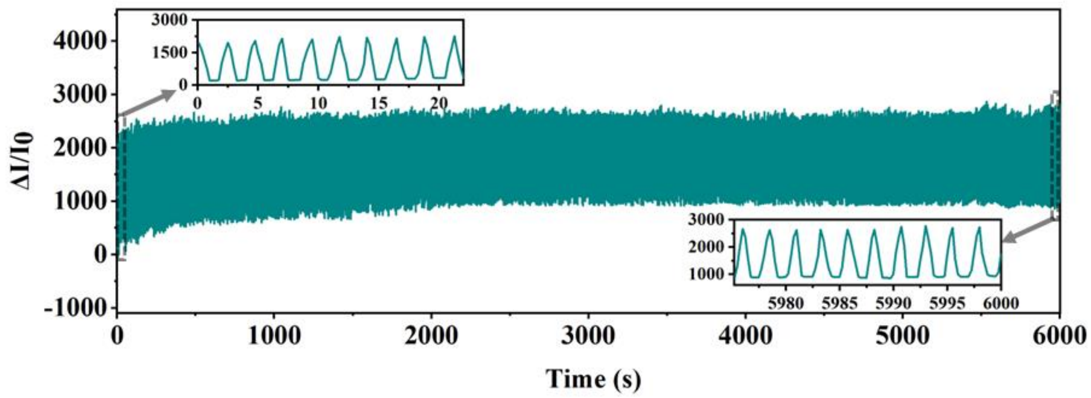

Fig. S12 Cycling performance of the DMWES working as pressure sensor at 10 kPa

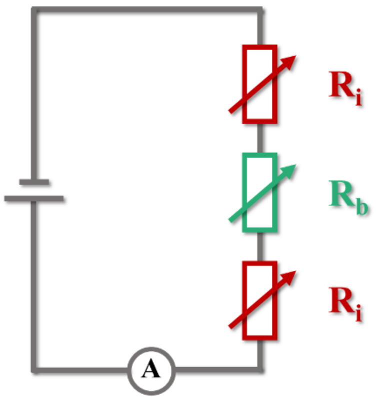

Fig. S13 Equivalent circuit of the resistance change of the DMWES

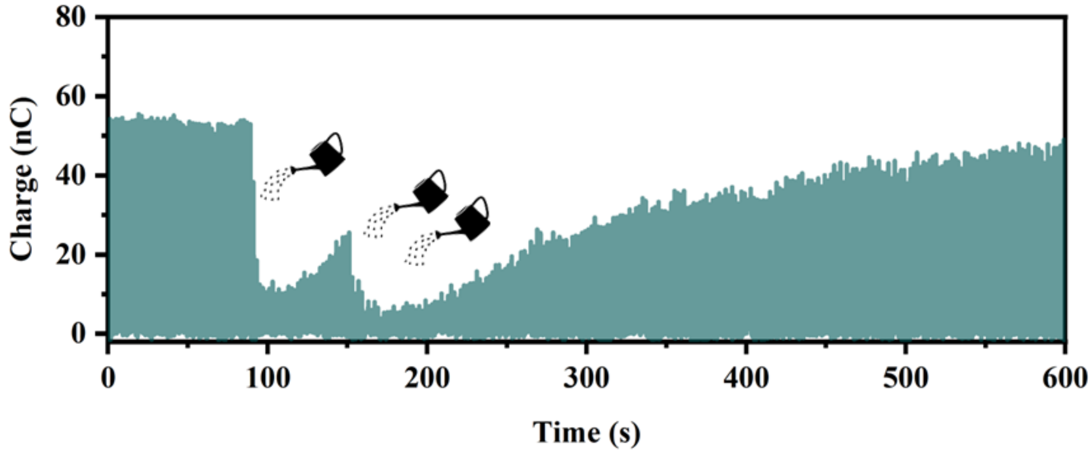

Fig. S14 Influence of water spraying treatment on the STENG performance

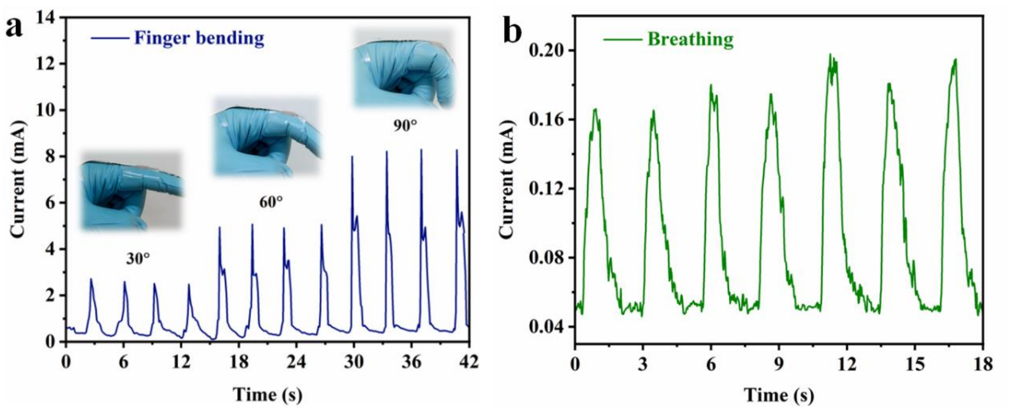

Fig. S15 (**a**) Finger bending and (**b**) breathing signal

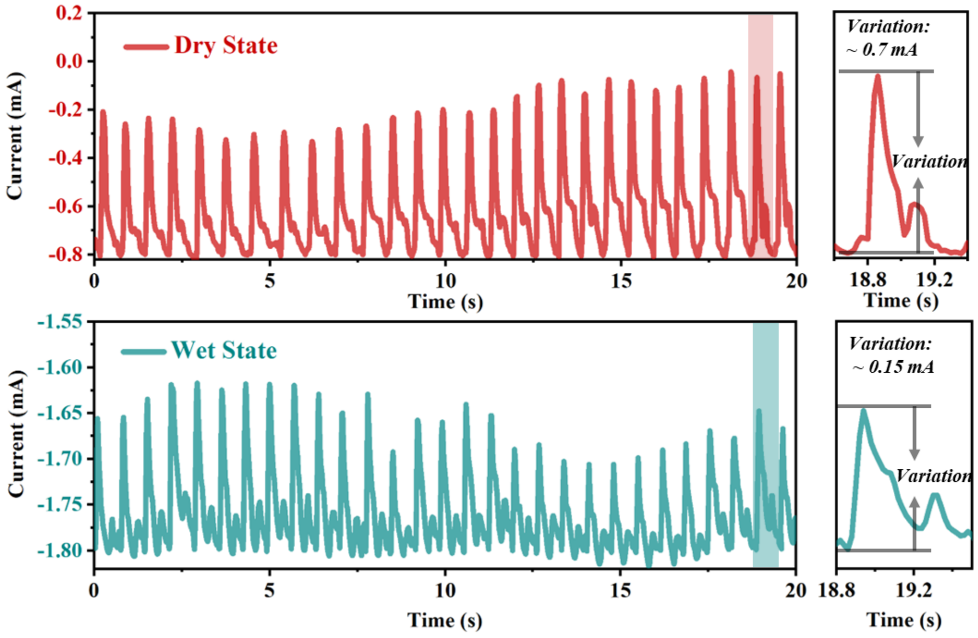

Fig. S16 Pulse monitoring performance of the DMWES in the dry state and the simulated sweat environment with NaCl solution

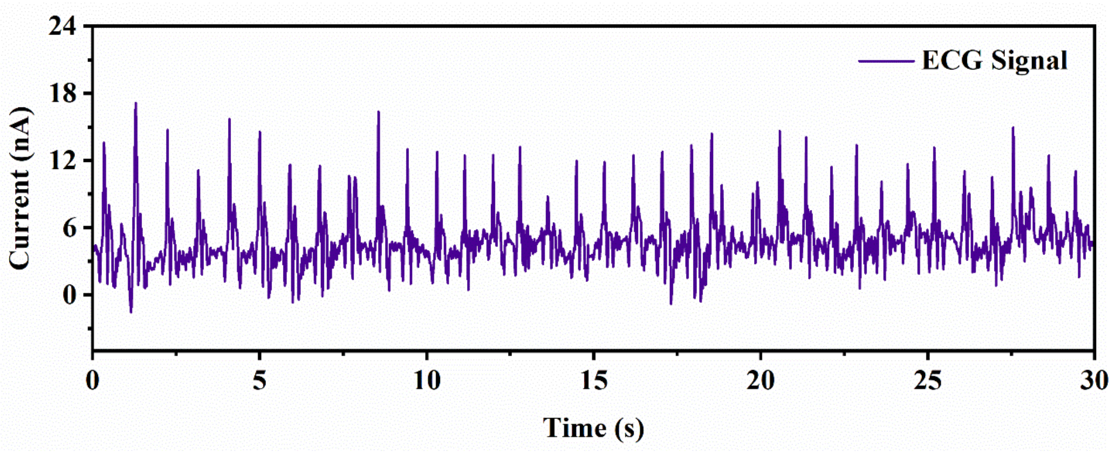

Fig. S17 ECG signal of the student 1

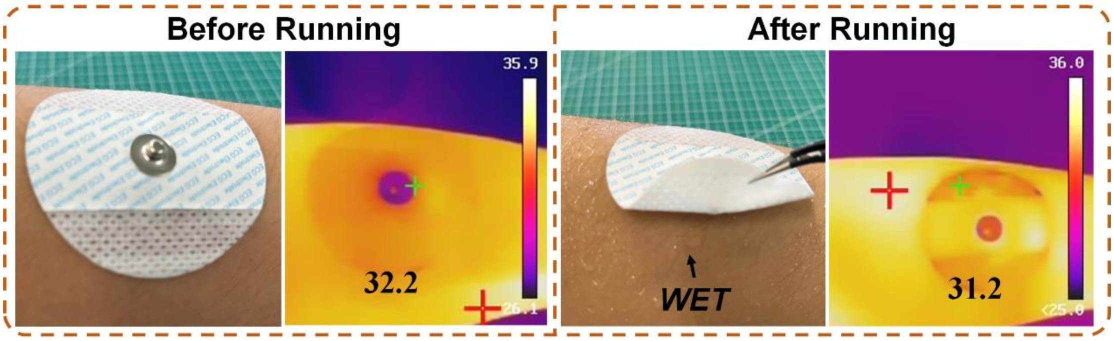

Fig. S18 Optical and Infrared camera images of the commercial gel electrode before and after running exercise

Table S1 Comparison on sensing performances between our work and the previous reports

Materials	Encapsulation	Detect limit (Pa)	Maximum sensitivity (kPa^{-1})	Maximum sensing range (kPa)	Response/ recovery time (ms)	Refs.
MXene/RGO Aerogel	PP	10	22.56	3.5	245/212	[S1]
MXene/PEDOT:PSS Aerogel	PDMS	--	26.65	11	106/95	[S2]
MXene-tissue paper	PLA	10.2	3.81	30	11/--	[S3]
accordion-like MXene	PET	9	99.5	4.3	4/13	[S4]
CS/MXene/PU sponge/PVA	Dust-free paper	50	140.6	22	200/30	[S5]
$Ti_3C_2T_x$@NWF	PDMS	--	6.31	150	300/260	[S6]
MXene/Bacterial Cellulose	PP	--	51.14	10.92	99/93	[S7]
PAN/ $Ti_3C_2T_x$	PET	1.5	104	8	30/20	[S8]
MXene/PDMS	PE	4.4	151.4	15	125/104	[S9]
MXene/PVA/PDMS	PDMS	0.88	403.46	18	105.3/99.3	[S10]
MXene/rGO/PS	PE	--	224	20	63/40	[S11]
CNT/$Ti_3C_2T_x$	PDMS	--	0.245	13	--/--	[S12]
C-PVDF/MXene-CNTs/PAN	Nonwoven Nanofibers	5	548.09	20	28.4/39.1	This work

Supplementary References

[S1] Y. Ma, Y. Yue, H. Zhang, F. Cheng, W. Zhao et al., 3D synergistical MXene/reduced graphene oxide aerogel for a piezoresistive sensor. ACS Nano **12**, 3209 (2018). https://doi.org/10.1021/acsnano.7b06909

[S2] S. Zhang, T. Tu, T. Li, Y. Cai, Z. Wang et al., 3D MXene/PEDOT:PSS composite aerogel with a controllable patterning property for highly sensitive wearable physical monitoring and robotic tactile sensing. ACS Appl. Mater. Interfaces **14**, 23877 (2022). https://doi.org/10.1021/acsami.2c03350

[S3] Y. Guo, M. Zhong, Z. Fang, P. Wan, G. Yu, A wearable transient pressure sensor made with MXene nanosheets for sensitive broad-range human–machine interfacing. Nano Lett. **19**, 1143 (2019). https://doi.org/10.1021/acs.nanolett.8b04514

[S4] Y. Gao, C. Yan, H. Huang, T. Yang, G. Tian et al., Microchannel-confined MXene based flexible piezoresistive multifunctional micro-force sensor. Adv. Funct. Mater. **30**, 1909603 (2020). https://doi.org/10.1002/adfm.201909603

[S5] X. Li, X. Li, T. Liu, Y. Lu, C. Shang et al., Wearable, washable, and highly sensitive piezoresistive pressure sensor based on a 3D sponge network for real-time monitoring human body activities. ACS Appl. Mater. Interfaces **13**, 46848 (2021). https://doi.org/10.1021/acsami.1c09975

[S6] Q. Yu, C. Su, S. Bi, Y. Huang, J. Li et al., Ti_3C_2Tx@nonwoven fabric composite: promising MXene-coated fabric for wearable piezoresistive pressure sensors. ACS Appl. Mater. Interfaces **14**, 9632 (2022). https://doi.org/10.1021/acsami.2c00980

[S7] T. Su, N. Liu, D. Lei, L. Wang, Z. Ren et al., Flexible MXene/bacterial cellulose film sound detector based on piezoresistive sensing mechanism. ACS Nano **16**, 8461 (2022). https://doi.org/10.1021/acsnano.2c03155

[S8] X. Fu, L. Wang, L. Zhao, Z. Yuan, Y. Zhang et al., Controlled assembly of MXene nanosheets as an electrode and active layer for high-performance electronic skin. Adv. Funct. Mater. **31**, 2010533 (2021). https://doi.org/10.1002/adfm.202010533

[S9] Y. Cheng, Y. Ma, L. Li, M. Zhu, Y. Yue et al., Bioinspired microspines for a high-performance spray Ti_3C_2Tx MXene-based piezoresistive sensor. ACS Nano **14**, 2145 (2020). https://doi.org/10.1021/acsnano.9b08952

[S10] J. Yan, Y. Ma, G. Jia, S. Zhao, Y. Yue et al., Bionic MXene based hybrid film design for an ultrasensitive piezoresistive pressure sensor. Chem. Eng. J. **431**, 133458 (2022). https://doi.org/10.1016/j.cej.2021.133458

[S11] L. Li, Y. Cheng, H. Cao, Z. Liang, Z. Liu et al., MXene/rGO/PS spheres multiple physical networks as high-performance pressure sensor. Nano Energy

95, 106986 (2022). https://doi.org/10.1016/j.nanoen.2022.106986

[S12] X. Zheng, Q. Hu, Z. Wang, W. Nie, P. Wang et al., Roll-to-roll layer-by-layer assembly bark-shaped carbon nanotube/Ti_3C_2Tx MXene textiles for wearable electronics. J. Colloid Interface Sci. **602**, 680 (2021). https://doi.org/10.1016/j.jcis.2021.06.043

Movie S1

The real-time directional water transport process of the DMWES.

Movie S2

The real-time pulse monitoring of the student 1 in dry and wet state via the fabricated DMWES.

Movie S3

The ECG monitoring of the student 1 via the wearable physiological monitoring system connecting the DMWES.

Movie S4

The directional water transport performance of the DMWES in the simulated sweat.
